# Proinflammatory transcriptomic and kinomic alterations in astrocytes derived from patients with familial Alzheimer's disease

**DOI:** 10.1016/j.bbih.2025.101044

**Published:** 2025-06-21

**Authors:** Benjamin Siciliano, Nicholas D. Henkel, William G. Ryan V, Ali Sajid Imami, John M. Vergis, Chongchong Xu, Taylen O. Arvay, Smita Sahay, Priyanka Pulvender, Abdul-rizaq Hamoud, Chadwick Hales, Robert E. McCullumsmith, Zhexing Wen

**Affiliations:** a The Graduate Program in Molecular and Systems Pharmacology, Emory University, Atlanta, GA, United States; b Department of Neurosciences and Psychiatry, University of Toledo College of Medicine and Life Sciences, Toledo, OH, United States; c Department of Psychiatry and Behavioral Sciences, Emory University School of Medicine, Atlanta, GA, United States; d Department of Neurology, Emory University School of Medicine, Atlanta, GA, United States; e Emory Goizueta Alzheimer's Disease Research Center, Emory University, Atlanta, GA, United States; f Center for Neurodegenerative Disease, Emory University, Atlanta, GA, United States; g Neurosciences Institute, ProMedica, Toledo, OH, United States; h Department of Cell Biology, Emory University School of Medicine, Atlanta, GA, United States; i Department of Human Genetics, Emory University School of Medicine, Atlanta, GA, United States

**Keywords:** Alzheimer's disease, Familial Alzheimer's disease, Astrocyte dysfunction, Multiomic analysis, Transcriptomics, Kinomics, Neuroinflammation, PI3K signaling, Protein kinase activity, Therapeutic targets in AD

## Abstract

Alzheimer's disease (AD) is a progressive neurodegenerative disorder characterized by profound neuronal and cognitive decline, with increasing evidence implicating astrocyte dysfunction in disease pathology. While traditional therapeutic approaches have primarily targeted neurons, the crucial role of astrocytes in metabolism, neurotransmission, amyloid-beta clearance, and neuroinflammation underscores their potential as therapeutic targets. In this study, we employed a multiomic integrative analysis combining transcriptomic and kinomic profiling of human induced pluripotent stem cell (hiPSC)-derived astrocytes from patients with familial AD (fAD) compared to healthy controls (HCs). Our transcriptomic analysis identified 1249 significantly differentially expressed genes, highlighting a pronounced upregulation of inflammatory genes (*SERPINA3*, *IL6R*, *IL1RAP*, *TNFRSF11A*) and a concomitant downregulation of genes essential for synaptic support and ion channel function (*STMN2*, *NMNAT2*, *SCN2A*, *GRIN1*). Kinomic profiling revealed dysregulated kinase activities within DYRK, GSK, and MAPK families, further implicating altered kinase signaling pathways in astrocyte dysfunction. Integration of these datasets pinpointed critical molecular hubs, notably within the PI3K signaling and inflammatory pathways, highlighting targets such as *JAK2*, *STAT3*, and *AKT1* as potential modulators of disease progression. Furthermore, leveraging the Library of Integrated Network-Based Cellular Signatures (LINCS) platform, we identified chemical perturbagens, including fluticasone propionate and Akt inhibitors, capable of reversing the transcriptomic signatures associated with fAD astrocytes. This integrative multiomic approach not only enhances our understanding of astrocyte-specific molecular mechanisms in AD but also provides novel targets for therapeutic intervention aimed at mitigating astrocyte-driven neurodegeneration.

## Highlights

• Familial AD astrocytes display significant pro-inflammatory transcriptomic and kinomic dysregulation.
• PI3K and inflammatory signaling pathways are highly dysregulated in familial AD astrocytes.
• Expression of inflammatory markers such as SERPINA3, IL6R, and TNFRSF11A is increased in familial AD astrocytes.
• Kinase activity analysis identifies DYRK, GSK, and MAPK pathways as key dysregulated axes in familial AD astrocytes.
• Potential astrocyte-specific therapeutic approaches to AD include targeting PI3K, JAK, and STAT3.

## 1 Introduction

Alzheimer's disease (AD), a leading cause of dementia, was the sixth leading cause of death in the United States in 2019 and the fifth leading cause of death in 2021 among individuals aged 65 and older (Alzheimer's disease facts and figures, 2024). Alzheimer's disease is a progressive neurodegenerative disorder characterized by significant neuronal damage that leads to cognitive decline, loss of function, and eventually death. The economic impact of AD is also substantial, with projected costs of $360 billion for 2024 (Alzheimer's disease facts and figures, 2024). Symptomatic medications for AD have been used for decades; however, their benefit is modest. Recently, amyloid monoclonal antibodies have been shown to demonstrate strong disease-modifying properties with amyloid removal and slowing of cognitive decline, but side effects that require close monitoring and efficacy for individual patients vary (Beshir et al., 2022; van Dyck et al., 2023; Sevigny et al., 2016; Sims et al., 2023; Placebo-Controlled, 2019; Assessment of Safety, 2020). Ongoing research and clinical trials continue to seek more effective treatments for both the disease and its symptoms, highlighting AD as a significant focus of global scientific efforts.

Astrocytes are critical to the pathophysiology of AD because of their role in metabolism, neurotransmission, and amyloid beta (Aβ) clearance (Beshir et al., 2022; van Dyck et al., 2023). For example, astrocytes upregulate EAAT2 to regulate neurotransmission and mitigate excitotoxicity (Carter et al., 2019; Rodríguez-Giraldo et al., 2022; Verkhratsky et al., 2010) while suppressing neuroinflammation through C3/STAT3 pathway downregulation (Takahashi et al., 2015). However, drug development efforts for neurodegenerative diseases have traditionally focused on neuronal targets, frequently neglecting the significant contributions of astrocytes to disease progression and pathology (Gorshkov et al., 2018). The development of astrocyte-specific therapeutic interventions that support astrocyte function could significantly advance the treatment of AD, offering new avenues to mitigate disease progression and improve patient outcomes.

Kinases orchestrate an array of cellular processes, including neuroinflammation in AD (Katafuchi et al., 2016; Rempe et al., 2010). Novel markers have been linked to neurotoxic astrocytes that drive AD progression, particularly those associated with proinflammatory responses (Toral-Rios et al., 2020; Reichenbach et al., 2019; Ceyzériat et al., 2018a). Previous transcriptomic studies have identified critical roles for astrocytes and protein kinases in AD pathogenesis. Specific mutations in protein kinases may drive cognitive decline in AD, independent of Aβ metabolism, load, and clearance (Yu et al., 2024; Galea et al., 2022; Serrano-Pozo et al., 2022). Notably, quantitative phosphoproteomic studies have revealed differential phosphorylation of protein kinases and small heat shock proteins (Xia et al., 2008; Ping et al., 2020; Dammer et al., 2015; Herskowitz et al., 2010). These studies also revealed increases in the phosphorylation of astrocyte-specific proteins such as GFAP, suggesting gliosis or astrocyte proliferation in AD (Dammer et al., 2015; Herskowitz et al., 2010). However, the kinase signaling pathways that specifically regulate astrocyte functions in AD remain poorly characterized. To enhance our understanding of AD and develop more effective interventions, it is crucial to delve deeper into astrocyte-specific kinases, phosphoproteins, and kinase-phosphosite networks in AD.

Familial AD (fAD), an early-onset form of AD, arises from autosomal-dominant mutations in genes such as *APP*, *PSEN1*, and *PSEN2* (D'Argenio et al., 2020). These mutations offer a genetically controlled model that is ideal for dissecting the molecular mechanisms underlying AD. Human induced pluripotent stem cell (hiPSC)-derived astrocytes from fAD patients may provide insights into astrocytic dysfunction and offer a platform for exploring astrocyte-specific therapeutic targets. In this study, we employed an integrative analysis of differential transcriptomic and kinomic signatures to identify molecular alterations associated with fAD, the hereditary form of AD, in hiPSC-derived astrocytes. We used RNA sequencing and PamGene kinomic profiling to identify key differentially expressed genes and dysregulated kinase activities in hiPSC-derived astrocytes from patients with fAD compared with age- and sex-matched HCs. The integration of RNA-seq and kinome array analyses provides a detailed view of both global gene expression changes and kinase activity alterations in AD. This combined approach enhances our understanding of the transcriptional changes and their functional implications in kinase signaling, specifically within astrocytes involved in AD progression. By correlating gene expression profiles with kinase activity, this methodology facilitates a more precise identification of potential therapeutic targets and contributes to deeper insight into the molecular mechanisms underlying astrocyte dysfunction in AD. The integration of these datasets enables the mapping of critical protein-protein interaction networks, uncovering central nodes and disrupted pathways that may serve as potential therapeutic targets. These integrative multiomic insights provide a deeper understanding of the molecular mechanisms underlying astrocyte dysfunction in fAD and suggest potential therapeutic targets for modulating disease progression.

## 2 Materials and methods

### 2.1 Culture of human iPSCs and differentiation into astrocytes

Three fAD human iPSC lines (AD-001: male, *APOE3/3*, *PSEN1* Y155H; AD-002: male, *APOE4/4*, *APP* V717I; AD-003: female, *APOE3/3*, *PSEN1* intron 4 deletion) and three HC human iPSC lines (HC-001: male, *APOE3/3*; HC-002: male, *APOE3/3*; HC-003: female, *APOE2/3*) were previously generated and fully characterized (Kuehner et al., 2021). The control and fAD subjects from which our hiPSCs were derived were matched for age and sex. For our analyses, fAD cells were compared with nonisogenic HCs. All experiments were performed in compliance with the relevant laws and institutional guidelines. hiPSCs were cultured in mTeSR1 medium (Stemcell Technologies, 85850) in cell culture dishes coated with Geltrex LDEV-Free Reduced Growth Factor Basement Membrane Matrix (Gibco, A1413201) diluted 1:100 in DMEM/F-12 (Gibco, 11320033). Briefly, mTesR1 was replaced every other day or every day once the cells reached 50 % confluence. When 80–90 % confluent, the cells were passaged as follows: aspirating media, washing with DPBS, incubating with ReLeSR (STEMCELL Technologies, 100–0484) at RT for 3 min, aspirating ReLeSR, incubating at 37 °C for 10 min, resuspending in mTeSR1 supplemented with 10 nM Y-27632 dihydrochloride ROCK inhibitor (Tocris, 125410), counting, and plating onto Matrigel-coated plates at the desired number.

Human iPSC-derived astrocytes were generated as previously described (Canals et al., 2018). Briefly, iPSCs were plated on Matrigel-coated 6-well plates and infected with *rtTA*, *SOX9*, or *NFIB* lentivirus. On Day 0, the medium was replaced with fresh mTeSR-1 media containing 2.5 μg/mL doxycycline. From Days 1–7, the cells were cultured in expansion medium (DMEM/F12, 10 % FBS, 1 % N2, 1.25 μg/mL puromycin, and 200 μg/mL hygromycin) and gradually transitioned to FGF medium (Neurobasal, 2 % B27, 1 % NEAA, 1 % GlutaMax, 1 % FBS, 8 ng/mL FGF, 5 ng/mL CNTF, 10 ng/mL BMP4, and 200 μg/mL hygromycin) by Day 7. On Day 7, the cells were dissociated via Accutase for 10 min and then replated on Matrigel-coated 6-well plates in FGF medium. From Day 9, the FGF medium was changed to maturation medium (1:1 DMEM/F12 and neurobasal medium, 1 % N2, 1 % Na pyruvate, 10 μg/μL NAC, 10 μg/μL hbEGF, 10 ng/mL CNTF, 10 ng/mL BMP4, and 500 μg/mL cAMP), and half of the medium was changed every 2–3 days.

For immunocytochemistry, astrocytes were fixed with 4 % paraformaldehyde for 15 min at room temperature. The samples were permeabilized and blocked with 0.25 % Triton X-100 and 10 % donkey serum in PBS for 20 min, as previously described (Wen et al., 2014). The samples were then incubated with anti-GFAP (rat, 1:500; Invitrogen 13–0300) and anti-S100B (mouse, 1:500; Sigma‒Aldrich AMAB91038) primary antibodies at 4 °C overnight, followed by incubation with secondary antibodies (donkey anti-rat Alexa Fluor 488, 1:1000; Invitrogen A-21208; donkey anti-mouse Alexa Fluor 568, 1:1000; Invitrogen A10037) for 2 h at room temperature. Coverslips were mounted with Fluoromount-G (Southern Biotech, 0100-01) and imaged via a Nikon Eclipse Ti-E microscope.

### 2.2 Sample preparation and processing for -omics studies

For RNA-seq, total RNA samples from the six cell lines were prepared with TRIzol reagent (Invitrogen). Total RNA was treated with DNase I (Zymo Research) and concentrated via the Clean & Concentrator Kit (Zymo Research). A NanoDrop One/OneC Microvolume UV–Vis Spectrophotometer (Thermo Scientific) was used to determine the RNA yield. For mRNA-enriched RNA-seq in all six cell lines, 150-cycle PE sequencing via the Illumina HiSeq2500 platform was performed with 1 μg of RNase-free RNA (>10 ng/μL, RIN >7) per technical triplicate. Transcript abundances were quantified from paired-end reads (raw FASTQ files) against the Homo sapiens reference transcriptome (hg19) via kallisto [360]. Transcriptome-wide gene counts were obtained through gene-level transcript summarization with the tximport R package [361]. Differential gene expression (DGE) analysis was performed after filtering for genes with low expression using the filterByExpr function via the edgeR R package [362].

For kinomics, each of the six iPSC samples (three HC and three fAD patients) was homogenized in mammalian protein extraction reagent (Thermo Fisher, 78501) containing 1 × Halt phosphatase and protease inhibitor cocktail (Thermo Fisher, 78443). The samples were incubated on ice for 30 min and subsequently run through the Fisherbrand Bead Mill 24 Homogenizer (Thermo Fisher, 15340163) for 5 min at 14000×*g*. The supernatant was collected and quantified via the Pierce BCA protein assay (Thermo Fisher, 23225). All the samples in each group were pooled according to total protein content and diluted with the same lysis buffer to 0.2 μg/μL. The six sample groups were then loaded on a single STK PamChip array (PamGene) and run in technical triplicate using three separate chips. Two micrograms of total protein were loaded into each array, followed by 30 min of blocking with 2 % bovine serum albumin (BSA), with a master mixture containing a final concentration of 90.91 μM adenosine triphosphate (ATP), 0.1 × BSA solution, 1 × protein kinase buffer, water, and a primary proprietary antibody mixture (PamGene). The sample and master mixture are incubated within the PamStation 12 (PamGene) for 80 min and pumped through the well to facilitate the phosphorylation of the immobilized peptides. In the subsequent step, FITC-labeled, anti-phosphoserine-threonine antibodies (PamGene) were added to each array. Each array was washed using PBS-T buffer (1 × PBS, 0.1 % Triton-X100), and Evolve2 (PamGene) kinetic image capture software was used to capture peptide phosphorylation on each PamChip. During the post-wash phase, the intensity of peptide spots was recorded at 10, 20, 50, 100, and 200 ms, then Bionavigator software was used to convert the captured images and their signal intensity to numerical values used for data analysis.

### 2.3 Transcriptomic analysis

The ggplot2 R package was used for volcano plots to depict differential expression profiles. The volcano plots display the difference in gene expression (log2FoldChange) between experimental comparisons and significance values (-log10 adjusted *p* value), with dashed lines indicating the significance thresholds of adjusted *p* values < 0.05 and absolute log2-fold change values > 1. For the heatmaps, the pheatmap R package was used, and the differentially expressed genes were filtered by AD risk-associated genes identified through Multimarker Analysis of GenoMic Annotation (MAGMA) of genome-wide association studies (GWAS) in late-onset AD (Rangaraju et al., 2018). Z scores were calculated via the scale function in R, ensuring normalized values for comparison across samples.

### 2.4 Transcriptomic pathway analysis

We utilized the 3PodReports package to conduct gene set enrichment analysis (GSEA) with the entire set of genes ranked by logFC and -log10 (p value) and overrepresentation analysis (ORA) of genes in the top and bottom 10 % of genes ranked by log2FoldChange [364, 365]. We then analyzed the overlap between GSEA and ORA.

### 2.5 Protein-protein interaction network analysis

To construct protein-protein interaction (PPI) networks, we used the Search Tool for the Retrieval of Interacting Genes/Proteins (STRING) for Homo sapiens to map differentiall expressed genes (DEGs) to known interactors. Significant DEGs (adjusted *p* value < 0.05, |logFC| > 1) were filtered for specific gene ontology (GO) terms, as annotated by the GO knowledgebase (Consortium et al., 2023; Ashburner et al., 2000), and STRING was used to retrieve interaction data for mapped genes (Szklarczyk et al., 2023). The network was visualized via igraph (Csárdi et al., 2006). Node sizes were scaled inversely to the adjusted *p* value, node colors were determined by logFC (red for upregulated genes and blue for downregulated genes), and the layout was generated via the Graphopt algorithm.

### 2.6 Transcription factor activity inference

Transcription factor (TF) activity was inferred from bulk RNA-seq data via the decoupleR package (Badia-i-Mompel et al., 2022). The input gene-level statistics, derived from differential gene expression analysis with edgeR, included t values and log2FC values for each gene. TF activities were inferred via the univariate linear model (ULM) method, which fits a linear model to predict observed gene expression on the basis solely of TF‒gene interaction weights from the CollecTRI network (Müller-Dott et al., 2023). The resulting t value from the fitted model was used as the TF activity score, where positive scores indicate TF activation and negative scores represent TF inhibition.

### 2.7 LINCS-based identification of discordant perturbagens

To identify small-molecule perturbagens that are predicted to reverse transcriptional alterations in astrocytes associated with fAD, we queried the Library of Integrated Network-based Cellular Signatures (LINCS). DEGs between fAD and HC conditions were used to generate lists of discordant mechanisms of actions (MOAs) and perturbagens. LINCS similarity scores were calculated to rank compounds on the basis of their potential to induce gene expression profiles opposing those observed in the fAD astrocytes. The top-ranked discordant MOAs were selected on the basis of the frequency (count) of compounds per MOA, and the top-ranked discordant perturbagens were selected on the basis of absolute similarity scores.

### 2.8 Baricitinib treatment of fAD iPSC-derived astrocytes

To investigate the effects of JAK/STAT inhibition on fAD-associated transcriptional changes, iPSC-derived astrocytes from fAD lines were treated with 200 nM baricitinib (Selleckchem, Catalog No. S2851) or vehicle (DMSO) for 72 h during days 25–28 of astrocyte maturation. RNA was extracted using TRIzol, followed by DNase I treatment and purification (Zymo Research). Raw RNA-seq reads were processed using the validated nf-core bulk RNA-seq pipeline (Ewels et al., 2020). Adapter sequences and low-quality bases were assessed with FastQC (Andrews, 2010) and trimmed using TrimGalore (Martin, 2011). Reads were aligned to the hg38 human reference genome via STAR (Dobin et al., 2013) and quantified with Salmon (Patro et al., 2017). Differential expression analysis (fAD-BAR vs. fAD-VEH) was performed using DESeq2 (Love et al., 2014), with significance thresholds of |log2FC| > 1 and adjusted p < 0.05. GO enrichment analysis was conducted using clusterProfiler, with redundant terms filtered by Jaccard similarity (<0.5).

### 2.9 Kinomic analysis

We utilized the BioNavigator software tool (PamGene) to preprocess kinome data, conduct image analysis, interpret output measurements, and visualize, store, and share results from the sample screen. The processed data yielded a list of differentially phosphorylated reporter peptides, which served as raw data for subsequent analyses. To increase confidence in predicting upstream kinases influencing substrate phosphorylation differences, we employed three distinct kinase substrate mapping tools, namely, a kinome random sampling analyzer (KRSA) as the primary analysis tool, PamGene upstream kinase analysis (UKA), and kinase enrichment analysis version 3 (KEA3). Using the Creedenzymatic R package, we converted the analysis outputs into percentile rankings, divided them into quartiles, and visually summarized the quartiles. High-confidence candidate kinases were those detected in the top quartile of the results from all three analyses, whereas medium-confidence candidates appeared in the top quartile of the KRSA results and at least the top two quartiles of the UKA and KEA3 results.

### 2.10 Multiomic integration

We employed the Kinograte R package, which utilizes an optimized version of the PCSF algorithm, to construct an integrated multiomic PPI network. This network included transcriptomic hits (FDR <0.05) and Creedenzymatic-predicted kinomic hits. Node prizes were assigned on the basis of the percentile rank of log2FoldChange or mean Creedenzymatic score, whereas edge costs were determined by the inverse STRING database interaction confidence. The PPI network nodes were subsequently ranked using the average of their node prize and eigencentrality for gene-set enrichment analysis with the fgsea R package, and the top 10 % of these nodes were also tested for overrepresentation analysis with the enrichR R package via Gene Ontology. Subnetworks of gene members from dysregulated pathways (FDR <0.05) identified in the primary PPI network were visualized. Pathways exhibiting dysregulation (FDR <0.05) were subjected to functional clustering and visualization via Pathway Analysis Visualization with Embedding Representations (PAVER), a meta-clustering method specifically designed for pathways (Ryan, 2023; Ryan et al., 2024). PAVER identifies the most representative terms (MRTs) for hierarchically clustered pathway embeddings by selecting the term with the highest cosine similarity to its respective cluster's average embedding. UMAP scatter plots were created, portraying individual pathways with colors indicating their cluster affiliation and shapes denoting the pathway analysis.

### 2.11 Sample size and statistical analyses

For RNA-seq, three fAD iPSC-derived astrocyte lines and three nonisogenic matched HC lines were used with three technical replicates each. For kinomics, three fAD iPSC-derived astrocyte lines and three nonisogenic matched HC lines were used with three technical replicates each. All genes and pathways analyzed had FDR <0.05 and log2FC > 1.

## 3 Results

### 3.1 Transcriptomic profiling reveals key dysregulated pathways in fAD astrocytes

**Fig. 1:**
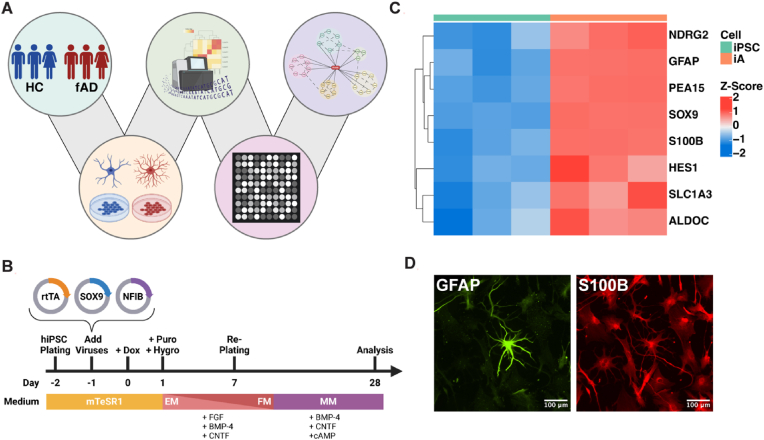
**Multiomics integration and characterization of hiPSC-derived astrocytes from fAD patients. (A)** Differential transcriptomic and kinomic signatures from iPSC-derived astrocytes from patients with fAD (n = 3) and age- and sex-matched nonisogenic HC individuals (n = 3) were subjected to network-based multiomics integration to identify dysregulated molecular pathways. (**B**) hiPSC-derived astrocytes were generated as previously described (Canals et al., 2018). (**C**) Heatmap showing the differential expression of canonical astrocyte markers in astrocytes relative to iPSCs. (**D**) Immunofluorescence staining of hiPSC-derived astrocytes at 20× magnification showing GFAP (green) and S100B (red) expression. HC: healthy control; fAD: familial Alzheimer's disease; iPSC: induced pluripotent stem cell; puro: puromycin; hygro: hygromycin; EM: expansion medium; FM: FGF medium; MM: maturation medium. (For interpretation of the references to color in this figure legend, the reader is referred to the Web version of this article.)

Our multiomics approach integrated differential transcriptomic and kinomic signatures from iPSC-derived astrocytes of fAD patients (n = 3) and matched HC individuals (n = 3) to identify dysregulated molecular pathways (Fig. 1A). Human iPSC-derived astrocytes were generated as previously described (Canals et al., 2018) (Fig. 1B). Differentiation was verified through RNA sequencing and immunocytochemistry. Heatmap analysis revealed increased expression of canonical astrocyte transcripts, including GFAP and S100B, in hiPSC-derived astrocytes compared with that in iPSCs (Fig. 1C). Immunofluorescence analysis further validated the astrocytic phenotype, with strong staining for GFAP and S100B (Fig. 1D). Following astrocytic differentiation, RNA was extracted from 3 familial AD iPSC-derived astrocyte lines and three nonisogenic matched control lines, and RNA sequencing was performed to investigate the molecular alterations in fAD astrocytes relative to controls.

**Fig. 2:**
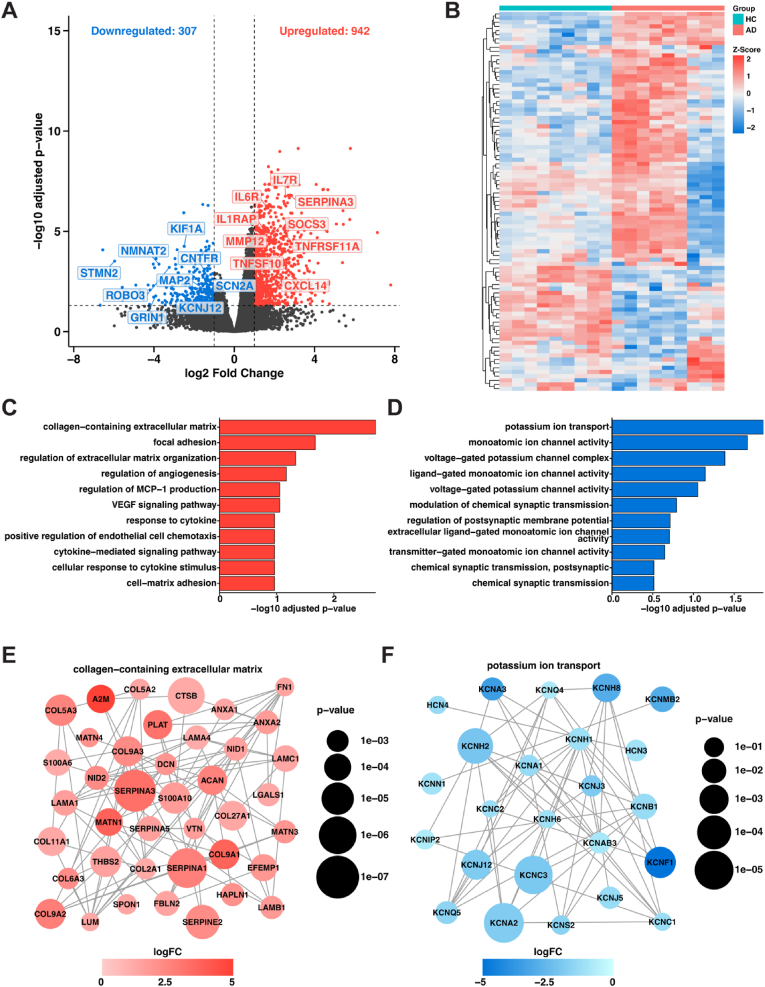
**Transcriptomic analysis of fAD astrocytes compared with HC astrocytes.** (**A**) Volcano plot of differential gene expression analysis comparing hiPSCs from HC and fAD astrocytes, plotted as a function of the log2-fold change in gene expression and the inverse log10 of the adjusted p value. The dashed lines demarcate adjusted p values less than 0.05 and absolute values of log2-fold change greater than 1. (**B**) Heatmap showing the differential expression of AD-associated GWAS hits in fAD astrocytes. (**C**) Top 10 upregulated and (**D**) top 10 downregulated pathways in fAD astrocytes identified via both rank-based GSEA and ORA. (**E**) PPI network of genes associated with “collagen-containing extracellular matrix” (GO:0062023) and (**F**) PPI network of genes associated with “potassium ion transport” network (GO:0006813), with the node color and size indicating the direction and significance of gene expression changes. Red nodes indicate significantly upregulated genes (log2-fold change >0, p < 0.05), whereas blue nodes represent significantly downregulated genes (log2-fold change <0, p < 0.05). The size of each node is proportional to the significance of the adjusted p value, with larger nodes representing more statistically significant changes in gene expression. HC: healthy control; fAD: familial Alzheimer's disease; AD: Alzheimer's disease; GWAS: genome-wide association study; GSEA: gene set enrichment analysis; ORA: overrepresentation analysis; PPI: protein-protein interaction. (For interpretation of the references to color in this figure legend, the reader is referred to the Web version of this article.)

To elucidate the molecular mechanisms underlying astrocyte dysfunction in fAD, a comprehensive transcriptomic analysis was conducted to compare the gene expression profiles of fAD astrocytes with those of HCs. This analysis identified 1249 significantly differentially expressed genes (adjusted p value < 0.05, |logFC| > 1). Among these genes, 307 were downregulated, and 942 were upregulated in fAD astrocytes compared with HCs. The transcript levels of genes related to the inflammatory response, synaptic function, and ion channel activity were significantly altered. Inflammatory response genes such as *SERPINA3*, *IL7R*, *IL6R*, *MMP12*, *IL1RAP*, TNFRSF11A, *TNFSF10*, and *CXCL14* were significantly upregulated, whereas synaptic function and ion channel activity genes such as *STMN2*, *NMNAT2*, *SCN2A*, *GRIN1*, and *KIF1A* were significantly downregulated (Fig. 2A). These results suggest a shift toward a more reactive, inflammatory state in fAD astrocytes, alongside a reduction in synaptic support functions. To investigate the relevance of transcriptomic signatures in fAD astrocytes, we determined whether the differentially expressed genes in our model were enriched for known AD genetic risk factors identified by GWAS analyses in humans. We probed the MAGMA study, a GWAS of late-onset AD, to determine whether our differentially expressed fAD genes were among those identified as AD risk-associated genes. The expression of *MEF2C*, *OAS1*, and *SORL1* was significantly altered, among other genes (Fig. 2B). The integration of GWAS analyses into our investigation validated the relevance of our fAD astrocyte model.

To elucidate the molecular dynamics in fAD astrocytes and their impact on disease progression, GSEA and ORA were conducted to identify pathways that are significantly altered (Ryan, 2023; Ryan et al., 2024). The pathways reported were present in both the GSEA and ORA results, thus providing greater confidence in their relevance to fAD astrocytes. The upregulated pathways included those related to the extracellular matrix, such as the “collagen-containing extracellular matrix” (GO:0062023) and “cytokine-mediated signaling pathways” (GO:0019221), suggesting enhanced inflammatory and structural remodeling in fAD astrocytes (Fig. 2C). On the other hand, downregulated pathways involve crucial neuronal functions, including “potassium ion transport” (GO:0006813), “synapse organization” (GO:0050808), and “voltage-gated potassium channel activity” (GO:0005249), which may contribute to the impaired neuronal communication observed in AD (Fig. 2D). Overall, these findings illustrate a multifaceted disruption in cellular pathways within fAD astrocytes, underscoring their potential role in exacerbating AD pathology through both inflammatory processes and neuronal dysfunction.

We then examined established PPI networks to explore how changes in gene expression affect functional cellular pathways in fAD astrocytes. The PPI network associated with the “collagen-containing extracellular matrix” (GO:0062023) revealed significant upregulation of numerous genes in fAD astrocytes (Fig. 2E). Key regulatory genes showing both high connectivity and high centrality include *FN1*, *ACAN*, *NID1*, and *LUM*, indicating their pivotal roles in ECM remodeling. The most significantly differentially expressed genes in this pathway include *SERPINA3* (logFC = 2.77), *CTSB* (logFC = 1.03), and *SERPINA1* (logFC = 2.32), all known to be involved in neuroinflammatory responses. Of particular interest is *A2M*, showing the highest fold change (logFC = 4.24) while maintaining substantial network connectivity. In contrast, the “potassium ion transport” network (GO:0006813) revealed significant downregulation in fAD astrocytes (Fig. 2F), with *KCNF1* exhibiting the highest fold change (logFC = 4.91) and *KCNA2* showing the most significant differential expression (p = 2.5e-04). *KCNA1* emerged as a key regulatory gene with high connectivity (22 connections) and centrality. Together, these findings suggest that fAD astrocytes are characterized by increased inflammation and structural remodeling coupled with diminished support for neuronal function, providing key insights into the cellular mechanisms driving AD pathology in astrocytes.

### 3.2 Identification of astrocyte-specific molecular targets in AD

**Fig. 3:**
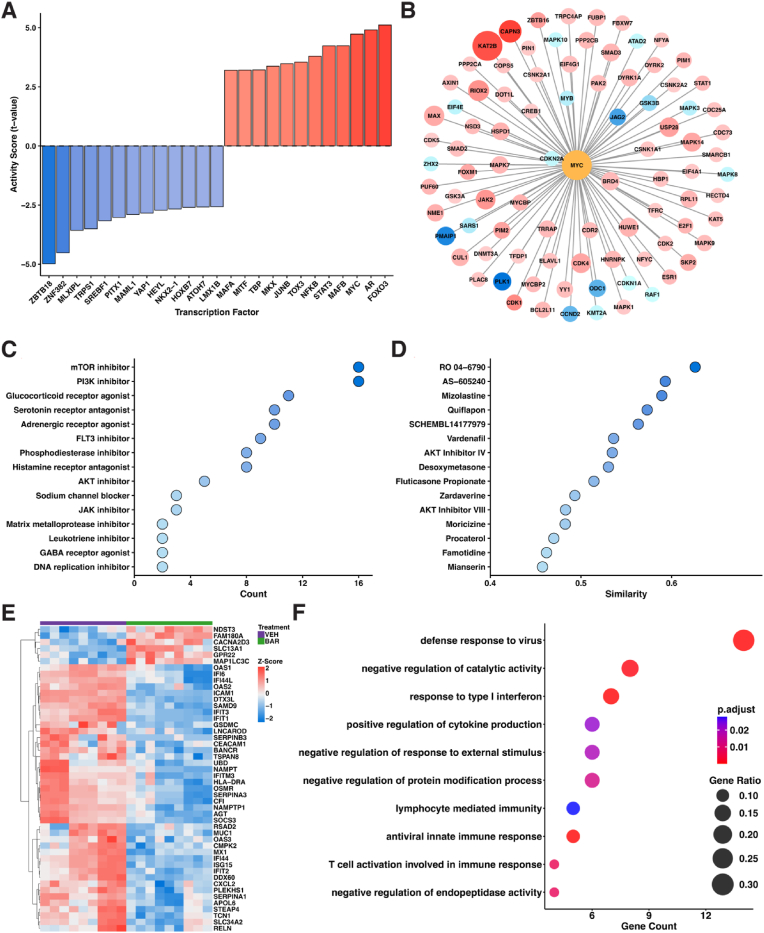
**Identification of astrocyte-specific molecular targets in fAD**. (**A**) The results of TF activity inference in fAD astrocytes vs HCs. Positive scores (red bars) indicate active TFs, whereas negative scores (blue bars) reflect inactive TFs. (**B**) A MYC-centered PPI network in which nodes represent genes interacting with MYC; red represents significantly upregulated genes (log2-fold change >1, p < 0.05), and blue represents significantly downregulated genes (log2-fold change < −1, p < 0.05). The size of each node is proportional to the significance of the adjusted p value, with larger nodes reflecting greater significance. (**C**) The discordant mechanisms of action identified through LINCS. The color of each point is proportional to the number of perturbations, with darker nodes reflecting a greater number of perturbations. (**D**) The top discordant perturbagens identified through LINCS. The color of each point is proportional to the similarity score, with darker nodes reflecting greater similarity. (**E**) Heatmap of DEGs in fAD astrocytes treated with baricitinib versus vehicle. Rows represent genes (|log2FC| > 1, adjusted p < 0.05), and columns represent biological replicates. Expression values are row-scaled (Z-scores), with red indicating upregulation and blue indicating downregulation relative to the mean. (**F**) Dot plot of GO biological processes enriched in BAR-treated fAD astrocytes. Dot size reflects the gene ratio, and color indicates the adjusted pvalue. Only non-redundant terms (Jaccard similarity <0.5) are shown. fAD: familial Alzheimer's disease; HC: healthy control; TF: transcription factor; PPI: protein-protein interaction; LINCS: Library of Integrated Network-Based Cellular Signatures; BAR, baricitinib; VEH, vehicle. (For interpretation of the references to color in this figure legend, the reader is referred to the Web version of this article.)

To elucidate the molecular mechanisms driving astrocyte dysfunction in fAD, an analysis of TF activity was conducted via the gene expression profiles of fAD astrocytes and HC astrocytes. This analysis aimed to identify the key transcription factors that are either activated or deactivated in fAD astrocytes, providing insight into their roles in the progression of AD pathology. TF activity was inferred via the ULM method, where t values were derived to predict the impact of TFs on gene expression (Badia-i-Mompel et al., 2022; Müller-Dott et al., 2023). Positive t values indicate activated TFs, reflecting increased target gene expression, whereas negative t values indicate deactivated TFs, which are associated with reduced gene expression. TF activity analysis revealed both activated and deactivated TFs in fAD astrocytes (Fig. 3A). Among the most significantly activated TFs were *FOXO3* (t = 5.116), *AR* (t = 4.906), and *MYC* (t = 4.730), which demonstrated both strong activation scores and extensive regulatory impact with high target gene counts (195, 512, and 886 targets, respectively). Other prominently activated TFs include *STAT3* (t = 4.232) and *NFKB* (t = 3.798), which are critical regulators of inflammation and cellular stress responses (Hu et al., 2014; Song et al., 2020; Koo et al., 2010; Liu et al., 2025; Peripolli et al., 2024; Ding et al., 2024). Conversely, the most strongly deactivated TFs included *ZBTB18* (t = −4.984), *ZNF382* (t = −4.518), and *MLXIPL* (t = −3.571). However, *SREBF1* emerged as particularly significant among the repressed factors due to its combination of strong deactivation (t = −3.164) and substantial regulatory influence (130 target genes). Other deactivated TFs such as *MAML1* and *YAP1* are implicated in cellular repair and metabolic regulation (Li et al., 2021; Shen et al., 2006; Fu et al., 2022), and their suppression suggests a loss of protective functions in fAD astrocytes. The deactivation of these TFs, particularly those with extensive target networks like *SREBF1*, may exacerbate cellular dysfunction and contribute to the progression of AD pathology in astrocytes.

To elucidate the molecular mechanisms underlying astrocyte dysfunction in fAD, *MYC* was selected as a central node for PPI analysis because it demonstrated strong activation (t = 4.73) in fAD astrocytes and the largest target network (886 target genes) among all assessed transcription factors (Fig. 3A). A *MYC*-centered PPI network was analyzed via interaction data retrieved from the OmniPath database (Türei et al., 2016) (Fig. 3B). The analysis identified *KAT2B* (logFC: 2.178), *CAPN3* (logFC: 2.500), and *BRD4* (logFC: 0.339) as highly significant interactors based on both statistical significance and network connectivity. Additionally, the network highlighted upregulation of key signaling molecules, including *JAK2* (logFC: 0.951), *CDK4* (logFC: 0.680), and *MAPK14* (logFC: 0.657). While *MYC* itself showed moderate upregulation (logFC: 0.432), its extensive connectivity (93 connections) underscores its potential role as a master regulator in fAD astrocytes. Targeting these key nodes, especially *MYC* and its highest-scoring interactors, presents viable therapeutic avenues to modulate transcriptional networks and restore cellular homeostasis in astrocytes, addressing core aspects of AD pathology.

To enhance our understanding of fAD and investigate potential therapeutic strategies, we leveraged the LINCS platform. This approach relies on log2-transformed fold changes and p-values from the LINCS L1000 project to pinpoint and categorize chemical perturbations based on their mechanisms of action (Subramanian et al., 2017; Lamb et al., 2006). Using this platform, we identified small molecules and their associated mechanisms of action that could potentially reverse the transcriptomic changes observed in fAD astrocytes compared to control astrocytes. Our analysis revealed key discordant MOAs that may counteract fAD-associated transcriptomic changes, including PI3K inhibitors (n = 16), mTOR inhibitors (n = 16), glucocorticoid receptor agonists (n = 11), adrenergic receptor agonists (n = 10), serotonin receptor antagonists (n = 10), FLT3 inhibitors (n = 9), histamine receptor antagonists (n = 8), phosphodiesterase inhibitors (n = 8), AKT inhibitors (n = 5), JAK inhibitors (n = 3), sodium channel blockers (n = 3), DNA replication inhibitors (n = 2), GABA receptor agonists (n = 2), leukotriene inhibitors (n = 2), and matrix metalloprotease inhibitors (n = 2) (Fig. 3C). Notable discordant perturbagens emerging from this analysis were RO 04–6790 (serotonin receptor antagonist; similarity = −0.626), AS-605240 (PI3K inhibitor; −0.593), mizolastine (histamine receptor antagonist; −0.589), quiflapon (leukotriene inhibitor; −0.573), SCHEMBL14177979 (tubulin inhibitor; −0.563), vardenafil (PDE5 inhibitor; −0.536), AKT Inhibitor IV (−0.535), desoxymetasone (glucocorticoid receptor agonist; −0.530), fluticasone propionate (glucocorticoid receptor agonist; −0.514), zardaverine (dual PDE3/PDE4 inhibitor; −0.493), AKT Inhibitor VIII (−0.483), moricizine (sodium channel blocker; −0.483), procaterol (adrenergic receptor agonist; −0.470), famotidine (histamine receptor antagonist; −0.462), and mianserin (multi-receptor antagonist; −0.458) (Fig. 3D). The prominence of PI3K/AKT/mTOR pathway modulators (AS-605240, AKT Inhibitor IV/VIII) alongside neuromodulatory compounds (mizolastine, mianserin, RO 04–6790) suggests multiple therapeutic avenues for reversing astrocytic transcriptomic dysregulation in fAD. This spectrum of discordant perturbagens, which includes anti-inflammatory agents (quiflapon, zardaverine), metabolic regulators (AKT inhibitors), and neurotransmission modulators (moricizine, procaterol), underscores the potential for targeting diverse astrocyte-driven pathways in AD pathology beyond conventional neuron-focused approaches.

Based on LINCS analysis predicting JAK/STAT inhibitors as top discordant perturbagens for reversing fAD-associated gene signatures (Fig. 3D), we performed a preliminary evaluation of baricitinib's effects in fAD astrocytes. Treatment with 200 nM baricitinib for 72 h yielded 48 differentially expressed genes (DEGs; adjusted p < 0.05, |log2FC| > 1), with predominant downregulation (42 downregulated vs. 6 upregulated) (Fig. 3E). Top DEGs included SERPINB3 (log2FC = −3.89) and ICAM1 (adjusted p = 8.48e-123), while GO analysis revealed enrichment in immune pathways, particularly "defense response to virus" (GO:0051607), "response to type I interferon" (GO:0034340), and "antiviral innate immune response" (GO:0140374) (Fig. 3F). These initial findings demonstrate that baricitinib, selected for its predicted transcriptional reversal potential, modulates neuroinflammatory signatures in fAD astrocytes.

### 3.3 Kinomic profiling reveals dysregulated kinase activity in fAD astrocytes

**Fig. 4:**
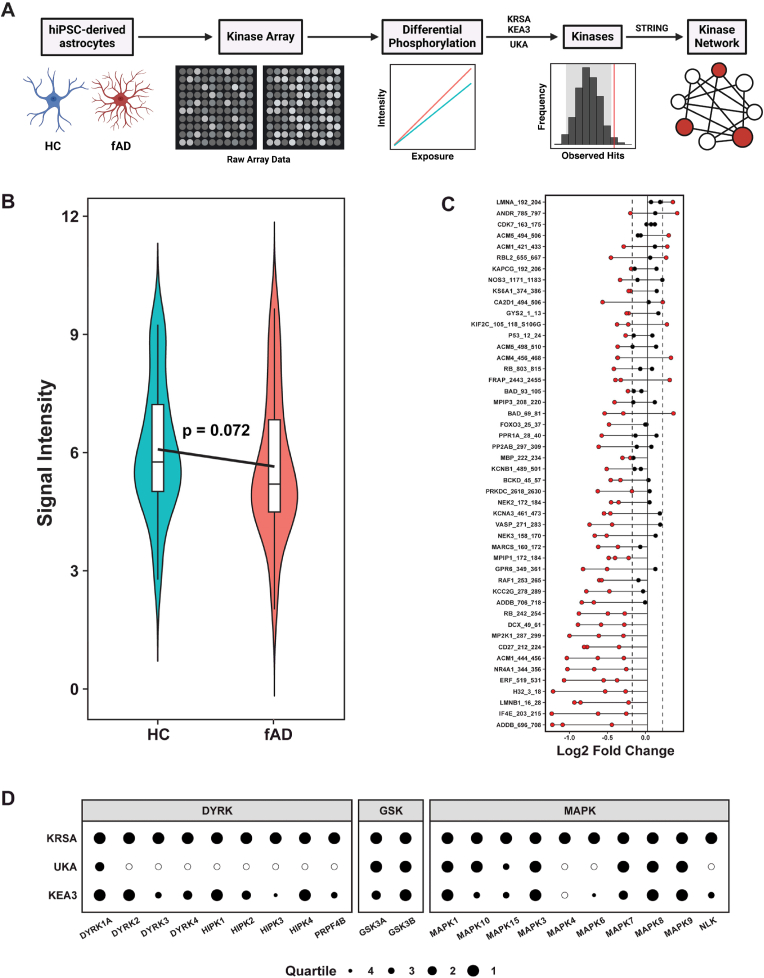
**Differential kinase activity in fAD astrocytes versus HC astrocytes.** (**A**) Peptides were initially selected on the basis of differences in phosphorylation between groups, and the upstream kinases that most likely drive these effects were identified via three different analyses of peptide phosphorylation. The emerging kinase hits were integrated into a larger kinase-to-kinase interaction network model. (**B**) Violin plot comparing global peptide phosphorylation (signal intensity) between fAD astrocytes and HCs. A two-tailed *t*-test revealed a trend toward reduced phosphorylation in fAD (p = 0.072). (**C**) Waterfall plot showing the log2 fold changes (x-axis) of phosphorylated peptides in fAD astrocytes compared to HCs, measured in triplicate (three independent kinome array chips). Each vertical line represents a peptide, with the highest absolute log2 fold change within each triplicate group highlighted by a horizontal line spanning from the maximum value to 0, passing through the other two replicates. Red filled circles indicate peptides with significant differential phosphorylation (log2 fold change < −0.2 or >0.2). Black filled circles denote peptides within the non-significant threshold. (**D**) Bubble plot integrating results from three kinase activity analysis platforms (KRSA, KEA3, UKA). Column headers represent kinase families, with individual kinases listed within columns. Black filled circles indicate kinases identified as active by the respective analyses (outlined circles: inactive/undetected). Circle size corresponds to the quartile ranking within each analysis (1: smallest, lowest confidence; 4: largest, highest confidence). iPSCs: induced pluripotent stem cells; HC: healthy control; fAD: familial Alzheimer's disease; KRSA: kinome random sampling analyzer; UKA: upstream kinase analysis; KEA3: kinase enrichment analysis 3. (For interpretation of the references to color in this figure legend, the reader is referred to the Web version of this article.)

To investigate the role of kinase dysregulation in fAD astrocytes, we employed the PamGene12 Kinome Array for comprehensive profiling of the human kinome (Fig. 4A). This platform quantifies phosphorylation patterns in biological samples through bioinformatic permutation, with rigorous validation of observed phosphopeptide signatures. Our analysis revealed altered phosphorylation patterns in fAD astrocytes compared with HCs. While a two-tailed *t*-test indicated that the reduction in global peptide phosphorylation (signal intensity) in fAD astrocytes did not reach statistical significance (p = 0.072) (Fig. 4B). Furthermore, specific peptides showed differential phosphorylation, suggesting targeted disruptions in signaling pathways (Fig. 4C). We employed three analytical modalities (KRSA, KEA3, UKA) to identify kinases driving these changes. To identify the kinases driving these changes, we employed three analytical modalities (KRSA, KEA3, UKA). All three methods converged on DYRK1A, GSK3A, GSK3B, and multiple MAPKs (MAPK1, MAPK3, MAPK7, MAPK8, MAPK9, MAPK10), with GSK3B, MAPK1, MAPK3, MAPK8, and MAPK9 ranked in the first quartile (highest confidence) across all platforms (Fig. 4D). Additional kinases, including DYRK2, HIPK1, HIPK4, and MAPK6, were identified by KRSA and KEA3, while MAPK4 was uniquely detected by KRSA (1st quartile). These kinases likely mediate the phosphorylation changes observed in Fig. 4C, as their activity directly influences the phosphorylation states of the peptides measured on the kinome array. Validation through a kinase interaction network model further underscored the centrality of these kinases to fAD pathology.

### 3.4 Multiomics integration highlights dysregulated pathways in fAD astrocytes

**Fig. 5:**
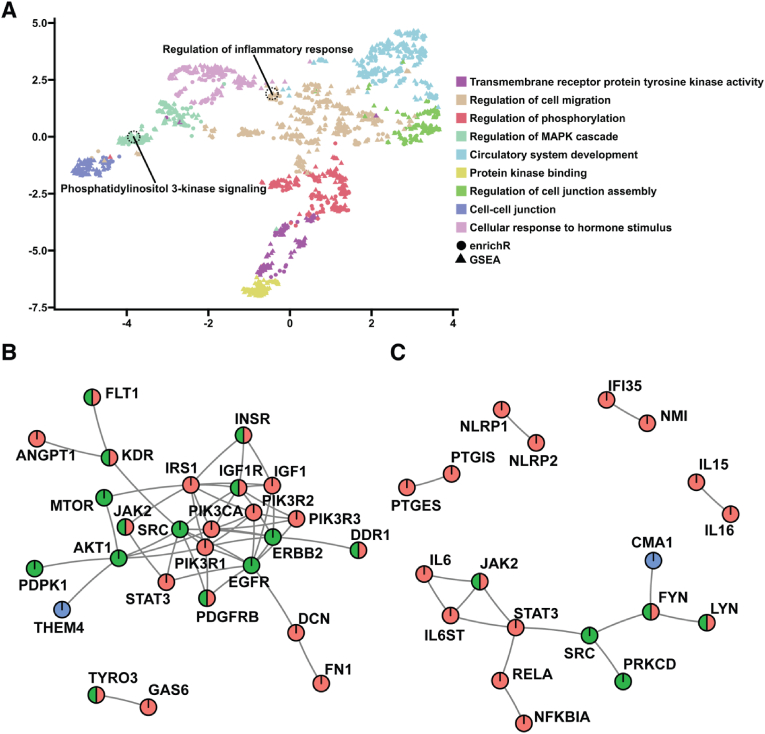
**Identification of molecular pathways altered in fAD astrocytes via integrative multiomics of the transcriptome and kinome.** (**A**) PAVER analysis of dysregulated molecular pathways revealed 10 functionally distinct gene set clusters in nonlinear UMAP space. (**B**) PPI network showing the subnetwork of genes involved in the PI3K pathway. (**C**) PPI network showing the subnetwork of genes involved in inflammatory pathways. Node color indicates omic hits, with red nodes indicating RNA, green nodes indicating kinases, and blue nodes indicating algorithmically interpolated hidden nodes. fAD: familial Alzheimer's disease; GSEA: gene set enrichment analysis; PAVER: pathway analysis visualization with embedding representations; UMAP: uniform manifold approximation and projection; PPI: protein-protein interaction. (For interpretation of the references to color in this figure legend, the reader is referred to the Web version of this article.)

To identify key dysregulated molecular pathways involved in astrocyte dysfunction and AD progression, we conducted an integrative multiomics analysis that combined transcriptomic and kinome array data. This integrated approach allows for a more comprehensive understanding of how changes in gene expression impact protein activity and signaling pathways in fAD astrocytes. Our analysis revealed significant dysregulation of key molecular pathways, particularly those involved in inflammatory responses and phosphatidylinositol 3-kinase (PI3K) signaling. The uniform manifold approximation and projection (UMAP) plot (Fig. 5A) visually represents the clustering of gene sets, highlighting distinct functional pathways, with notable emphasis on the regulation of inflammatory responses and PI3K signaling. These pathways were identified as critical areas of dysregulation in fAD astrocytes. PPI network analysis further elucidated these findings by mapping the interactions within these pathways. PPI network analysis of proteins in the PI3K signaling network revealed key nodes, such as *PIK3R1*, *IGF1*, and *AKT1*, which are central to this pathway and show substantial changes in connectivity and activity in fAD astrocytes (Fig. 5B). Similarly, PPI network analysis of proteins in the inflammatory response network highlighted the central roles of *IL6*, *JAK2*, and *STAT3* in mediating the inflammatory processes that are upregulated in the disease state (Fig. 5C). The integrative approach of this analysis revealed multiple multiomic hits in both networks, such as *JAK2*, indicating both transcriptomic dysregulation and kinomic changes in these targets. These results provide a comprehensive view of the molecular mechanisms underlying astrocyte dysfunction in AD, pinpointing specific pathways that may serve as potential therapeutic targets.

## 4 Discussion

This study provides valuable insights into the molecular landscape of astrocyte dysfunction in fAD through an integrative multiomic approach. Our transcriptomic analysis revealed a distinct inflammatory phenotype in fAD astrocytes, characterized by upregulation of inflammatory genes (*SERPINA3*, *IL6R*, *IL1RAP*, TNFRSF11A) and downregulation of genes involved in synaptic function and neuronal support (*STMN2*, *NMNAT2*, *SCN2A*, *GRIN1*, KIF1A). Pathway analysis confirmed enhanced inflammatory signaling and extracellular matrix remodeling, coupled with diminished potassium ion transport and synapse organization. Transcription factor activity analysis identified key molecular drivers, with inferred activation of *STAT3*, *NFKB*, and *MYC* and deactivation of *SREBF1*, *MAML1*, and *YAP1*. Complementing these findings, our kinomic analysis revealed significant alterations in kinase activity, particularly in the DYRK, GSK, and MAPK families, which are known to regulate neuroinflammation and synaptic function. The integration of these multiomics datasets through network-based approaches identified critical molecular hubs, including *PIK3R1*, *IGF1*, *AKT1*, *IL6*, *JAK2*, and *STAT3*, which represent promising therapeutic targets for addressing astrocyte dysfunction in fAD.

The inflammatory gene signature we observed in fAD astrocytes aligns with multiple studies highlighting the role of astrocyte-mediated neuroinflammation in AD pathogenesis. SERPINA3 upregulation, which we identified in our transcriptomic analysis, has been previously associated with senescent astrocytes, leading to increased proinflammatory factors and decreased neurotrophic support (Han et al., 2024; Liu et al., 2023; Vanni et al., 2017; Fissolo et al., 2021; Zattoni et al., 2022). Similarly, our finding of *IL6R* upregulation corresponds with research showing that the p.D358A variant increases soluble IL-6R levels, enhancing IL-6 trans-signaling in astrocytes and contributing to neuroinflammatory processes, particularly in *APOE4* carriers (Haddick et al., 2017; Quillen et al., 2023). Similarly, *IL1RAP* genetic variants correlate with accelerated amyloid accumulation, elevated cerebrospinal fluid (CSF) tau, and cognitive decline in AD cohorts (Zettergren et al., 2019; Ramanan et al., 2015), further implicating inflammatory pathways in disease progression. *GPR17*, another key mediator in our dataset, has been shown to drive neuroinflammatory responses to Aβ aggregates; its inhibition rescues cognitive deficits, suppresses neuroinflammation, and restores synaptic plasticity via modulation of NF-κB, Nrf2/HO-1, and BDNF pathways (Jin et al., 2021; Liang et al., 2023). Collectively, these findings position astrocytic inflammatory dysregulation as a critical nexus in AD, revealing molecular targets for disease-modifying strategies.

The diminished neuronal support we observed through the downregulation of synaptic function genes complements existing literature on astrocyte dysfunction in AD. *STMN2*, which we found downregulated in fAD astrocytes, has been identified as a key target of TDP-43 that undergoes aberrant splicing in AD patients (Krus et al., 2024). Furthermore, our finding of reduced *NMNAT2* expression parallels previous work showing that *NMNAT2* overexpression reduces Aβ production and tau phosphorylation in Tg2576 mice (Cheng et al., 2013, 2021), suggesting that its downregulation in astrocytes may contribute to AD pathology. Notably, our model recapitulates altered expression of GWAS-validated AD risk genes, including *MEF2C*, *OAS1*, and *SORL1* (Tansey et al., 2018; Salih et al., 2019; Miyashita et al., 2013), underscoring its translational relevance for probing AD pathophysiology.

Pathway analysis further delineated this dual astrocytic phenotype: upregulated extracellular matrix remodeling and cytokine signaling (Fig. 2C) coincided with downregulated potassium ion transport and synapse organization pathways (Fig. 2D). *FN1*, *ACAN*, *NID1*, and *LUM* showed both high connectivity and centrality in our PPI network analysis, suggesting their pivotal roles in pathological ECM remodeling (Fig. 2E). In contrast, *KCNA1* emerged as a critical regulatory hub with high connectivity and centrality within the potassium ion transport network. These results reveal astrocytes' dual role in AD pathogenesis, promoting neuroinflammation through ECM remodeling while simultaneously showing impaired potassium channel function, which disrupts ionic homeostasis and compromises neuronal communication (Liddelow et al., 2017; Akiyama et al., 2000), ultimately accelerating disease progression through multiple converging pathways.

Our transcription factor analysis identified *STAT3* activation, which aligns with studies demonstrating that *STAT3* pathway activation in astrocytes increases proinflammatory cytokines, exacerbates Aβ deposition, and impairs neuronal function (Toral-Rios et al., 2020; Kim et al., 2022). Conversely, *STAT3* inhibition has been shown to reduce neuroinflammation, decrease Aβ levels, and improve cognitive function in AD models (Reichenbach et al., 2019; Choi et al., 2020; Ben Haim et al., 2015; Ceyzériat et al., 2018b; Zhou et al., 2024; Mehla et al., 2021). Notably, we observed strong activation of *MYC* with an extensive regulatory network of 886 target genes, positioning it as a master regulator in fAD astrocytes. This aligns with studies showing upregulation of *MYC* proteins in reactive astrocytes across neurodegenerative diseases, contributing to astrogliosis independent of neuronal death (Ferrer et al., 2000). The extensive connectivity of *MYC* in our PPI network underscores its role in astrocyte dysfunction through cell cycle dysregulation, a mechanism implicated in Aβ-induced neuronal apoptosis (Lee et al., 2009, 2011). Additionally, our finding of *SREBF1* deactivation is consistent with reports of its downregulation in AD oligodendrocytes, contributing to neurodegeneration through disrupted cholesterol synthesis (Morabito et al., 2021; Mohamed et al., 2018). *YAP1* deactivation in our model corresponds with research showing that its loss promotes astrocyte senescence and contributes to neurodegenerative processes in AD (Xu et al., 2021a). We also identified significant activation of *FOXO3*, contrasting with reports of declining *FOXO3* levels in aging cortex and its protective role against Aβ pathology (Du et al., 2021). Our analysis identified several transcription factors with no previous association with Alzheimer's disease in astrocytes, including *TOX3*, *MKX*, *ZBTB18*, and *ZNF382*.

The kinomic changes we observed, particularly in DYRK, GSK, and MAPK family kinases, support previous studies implicating these kinases in AD pathophysiology. *DYRK1A* and *GSK3B* influence tau phosphorylation and neuroinflammatory responses (WOODS et al., 2001; Sayas et al., 2021), while MAPK signaling mediates cellular stress responses and inflammation (Saha et al., 2020; Webster et al., 2006). Our findings using the PamGene platform provide further evidence for the involvement of these kinase families in astrocyte dysfunction in fAD. The integration of our kinomic and transcriptomic data identified molecular hubs in the PI3K signaling pathway (*PIK3R1*, *IGF1*, *AKT1*) and inflammatory response pathway (*IL6*, *JAK2*, *STAT3*), which have been previously implicated in AD pathophysiology (Heneka et al., 2015; Calsolaro et al., 2016). Notably, our finding of *JAK2* dysregulation across multiple omics platforms validates its critical role in fAD pathology and aligns with existing literature on JAK-STAT signaling in neuroinflammation. The direct interactions between MYC and several key kinases in our network analysis position it as a potential master regulator of kinase dysregulation in AD pathophysiology. Specifically, MYC directly interacts with *MAPK14* (p38 MAPK) and *JAK2*, both significantly upregulated in our dataset, suggesting MYC-mediated dysregulation of stress-response and inflammatory signaling pathways.

Our LINCS analysis identified several small molecules with the potential to reverse the molecular alterations observed in fAD astrocytes. Discordant perturbagens such as fluticasone propionate, Akt inhibitor VIII, Akt inhibitor IV, and AS−605240 align with our pathway analysis findings and may offer therapeutic potential by targeting the inflammatory phenotype of fAD astrocytes. The strong representation of PI3K/AKT/mTOR pathway modulators (16 PI3K inhibitors, 16 mTOR inhibitors, and 5 AKT inhibitors) in our analysis highlights this signaling axis as a central therapeutic target. Our LINCS analysis identified modulation of JAK/STAT signaling as a top discordant mechanism for reversing fAD-associated gene signatures (Fig. 3D). Supporting this finding, baricitinib treatment in fAD astrocytes induced significant downregulation of immune-related transcripts (42/48 DEGs), including antiviral effectors (OAS2, MX1), interferon-stimulated genes (IFIT3, IFI44), and inflammatory mediators (SOCS3, ICAM1) (Bizzotto et al., 2020; Verhelst et al., 2012; Chikhalya et al., 2021; DeDiego et al., 2019; Chaves de Souza et al., 2013; Zhang et al., 2016). Pathway analysis confirmed enrichment of immune processes ("defense response to virus" [GO:0051607], "response to cytokine" [GO:0034097]), consistent with JAK/STAT's role in inflammatory signaling. While these transcriptional changes suggest potential for modulating astrocyte-mediated neuroinflammation, further studies are needed to determine functional consequences on neuronal viability and disease-relevant phenotypes.

The identification of fluticasone propionate is particularly promising, as previous studies have shown an association between fluticasone use and reduced risk of AD (Xu et al., 2021b; Lehrer et al., 2018; Zang et al., 2023). Intranasal delivery may provide more direct access to affected brain regions compared to oral corticosteroids, which have shown limited efficacy in previous clinical trials (Lehrer et al., 2018). Similarly, the adrenergic receptor agonist procaterol identified in our analysis has significant preclinical evidence supporting its therapeutic potential. Studies have shown that β3AR agonists improve recognition memory and reduce hippocampal Aβ42/Aβ40 ratios in AD mouse models (Tournissac et al., 2021), effects that mirror the potential benefits of LINCS-identified procaterol, which has activity at both β2 and β3 receptors. Chronic β3-adrenergic receptor (β3AR) activation has also been shown to enhance neurovascular coupling and reduce blood-brain barrier leakage in aged mice (Natarajan et al., 2025) Additionally, novel α1AR agonists have shown improved synaptic plasticity in AD models without hypertensive effects (Papay et al., 2023), addressing a key limitation of earlier adrenergic therapies.

Our findings regarding PI3K/AKT/mTOR pathway inhibitors parallel work with rapamycin, which has shown promise in targeting aging-related and metabolic aspects of AD pathology (Spilman et al., 2010; Harrison et al., 2009). Similarly, the anti-inflammatory compounds identified in our analysis share mechanistic overlap with PPAR-γ agonists like pioglitazone, which have been investigated in AD clinical trials (Fang et al., 2022). The Akt inhibitor VIII, which we identified, has been shown to reduce lipopolysaccharide (LPS)-induced MMP-9 overexpression, implicated in extracellular matrix breakdown and blood-brain barrier disruption in neuroinflammatory diseases (Yang et al., 2020). These small molecules, along with other identified compounds like SCHEMBL14177979 (tubulin inhibitor) that may address microtubule destabilization in AD (Duncan et al., 2013), could be further explored for their ability to modulate the dysregulated genes and kinases identified in this study. The breadth of identified compounds suggests that combination therapies targeting multiple astrocyte-driven pathways may be more effective than single-target approaches in addressing the complex pathophysiology of AD. Unlike traditional amyloid-focused therapies that target primarily neuronal pathways, our LINCS analysis emphasizes astrocyte-driven mechanisms through multiple complementary pathways, presenting opportunities for novel therapeutic interventions.

Our findings on astrocyte-specific molecular alterations in fAD present a distinct profile when compared with neuron and microglia-specific changes in AD models. While neurons primarily display dysregulation in tau phosphorylation pathways and GSK3β hyperactivity, with *APP* and *PSEN1/2* mutations driving Aβ production and synaptic dysfunction (Zhao et al., 2020; Penney et al., 2020; Ochalek et al., 2017), our astrocyte models reveal a predominant inflammatory signature characterized by upregulation of *SERPINA3*, *IL6R*, and *IL1RAP* alongside ECM remodeling genes (*FN1*, *ACAN*, *NID1*). Unlike neurons, which exhibit REST transcription factor perturbation (Meyer et al., 2019), astrocytes show STAT3, NFKB, and MYC activation paired with SREBF1 and YAP1 deactivation, suggesting cell-type specific transcriptional programs in AD pathogenesis. In contrast to microglia, which demonstrate *TREM2*-dependent disease-associated microglial (DAM) states with *APOE*/*SPP1* upregulation and p38 MAPK (*MAPK14*) activation (Reich et al., 2021; Dolan et al., 2023; Dräger et al., 2022), our fAD astrocytes exhibit dysregulation in complementary but distinct kinase families, particularly DYRK, GSK, and conventional MAPK. While all three cell types show altered PI3K/AKT pathway signaling, astrocytes uniquely display pronounced cytokine receptor upregulation (*IL6R*, *IL1RAP*) alongside reduced potassium ion transport and synaptic support functions, highlighting their dual role in neuroinflammation and loss of homeostatic support.

Notably, our kinomic analysis revealed substantial overlap with previously identified kinase alterations in neurons, particularly within the GSK family that drives tau hyperphosphorylation (Ochalek et al., 2017), but also identified astrocyte-specific signatures in JAK/STAT signaling that were not prominent in neuronal models. This contrasts with microglial models, where CSF1R kinase activity modulates SPP1-positive inflammatory states (Dräger et al., 2022). Our identification of multiple potential therapeutic compounds targeting astrocyte-specific pathways, including AS-605240 (PI3K inhibitor), RO 04–6790 (serotonin receptor antagonist), mizolastine (histamine receptor antagonist), fluticasone propionate (glucocorticoid receptor agonist), and procaterol (adrenergic receptor agonist), differs from approaches targeting neuronal GSK3β inhibitors or microglial *TREM2* enhancement. The prominence of anti-inflammatory agents like quiflapon (leukotriene inhibitor) and zardaverine (PDE3/PDE4 inhibitor) in our analysis emphasizes the unique inflammatory signature of astrocytes compared to other cell types in AD pathogenesis. These findings underscore the value of cell-type specific approaches to AD, suggesting that optimal therapeutic strategies may require targeting distinct pathways across multiple cell types to address the complex pathophysiology of this disease.

Despite the comprehensive insights gained from our multiomic approach, several limitations related to functional validation should be acknowledged. While our LINCS analysis identified promising therapeutic compounds, we did not functionally validate these in cellular or animal models of AD. Direct testing of compounds such as fluticasone propionate and β3-adrenergic receptor agonists on fAD astrocytes would provide critical evidence of their ability to reverse the inflammatory phenotype we observed. Furthermore, the computational nature of LINCS correlations requires verification through targeted mechanistic studies to confirm the predicted mode of action for each compound. The translational relevance of our findings is also limited by our use of familial AD astrocytes, which may not fully represent the pathophysiology of sporadic AD, the more common form of the disease. Finally, our *in vitro* model lacks the complex cellular interactions present in the brain, including crosstalk between astrocytes, neurons, microglia, and other cell types that contribute to AD pathogenesis, potentially affecting the efficacy of the therapeutic compounds identified in our analysis when tested in more complex systems.

Future research should prioritize functional validation of the molecular targets and therapeutic compounds identified in this study. For the LINCS-predicted compounds, we propose a systematic validation pipeline beginning with *in vitro* testing on fAD astrocytes to verify their ability to normalize the inflammatory phenotype and restore neuronal support functions. Novel compounds with strong discordance scores, such as serotonin receptor antagonist RO 04–6790 and histamine receptor antagonist mizolastine, deserve special attention as they represent potentially groundbreaking therapeutic approaches that target neuroinflammatory mechanisms through pathways distinct from traditional anti-inflammatory agents. For tubulin inhibitors like SCHEMBL14177979, testing should focus on their ability to stabilize microtubules in astrocytes and the subsequent effects on astrocyte-neuron interactions. This should include measuring the compounds' effects on key dysregulated pathways identified in our study, particularly STAT3 signaling, PI3K/AKT activity, and specific kinases in the DYRK, GSK, and MAPK families. The most promising candidates should then be advanced to co-culture systems with neurons to assess their ability to mitigate astrocyte-mediated neuronal damage. For lead compounds like fluticasone propionate and adrenergic receptor agonists, structure-activity relationship studies could optimize their efficacy and blood-brain barrier penetration. Beyond compound testing, genetic validation of key molecular hubs identified in our network analysis is essential. Genetic and pharmacological modulation of prioritized targets like *STAT3*, *PIK3R1*, and *JAK2*, followed by functional assays of inflammatory responses, metabolic function, and neuroprotective capacity, would confirm their causal roles in astrocyte dysfunction. This multi-pronged approach will distinguish direct effects from compensatory adaptations while identifying optimal therapeutic strategies.

## 5 Conclusions

In conclusion, this study elucidates the molecular alterations underlying astrocyte dysfunction in fAD through a comprehensive multiomic approach. By integrating transcriptomic and kinomic analyses, we identified critical dysregulated pathways driving fAD pathophysiology in astrocytes. Our findings highlight several particularly promising therapeutic targets: the STAT3 signaling axis, which showed consistent dysregulation across multiple analytical modalities and central positioning in inflammatory networks; the PI3K/AKT pathway, which emerged as a critical hub in our network analysis with strong connections to both metabolism and inflammatory signaling; and specific kinases in the DYRK, GSK, and MAPK families that demonstrated altered activity in our kinomic analysis and have been previously implicated in neuroinflammatory processes in AD. The pronounced inflammatory phenotype of fAD astrocytes, characterized by upregulation of *SERPINA3*, *IL6R*, and *IL1RAP*, alongside ECM remodeling genes like *FN1*, *ACAN*, and *NID1*, represents a distinct therapeutic opportunity compared to neuron-focused approaches. The emergence of *MYC* as a master regulator with extensive connectivity underscores its potential as a key target for modulating transcriptional networks in AD. The identification of chemical perturbagens that target these specific pathways, particularly fluticasone propionate and β3-adrenergic receptor agonists, provides concrete avenues for therapeutic intervention. These compounds show particular promise as they target the astrocyte-specific molecular abnormalities revealed in this study while also having established safety profiles that could expedite clinical development. These findings not only enhance our understanding of the molecular mechanisms contributing to AD but also provide a focused foundation for developing targeted therapies aimed at modulating astrocyte function while addressing the limitations identified in our study and pursuing the future directions outlined above.

## CRediT authorship contribution statement

**Benjamin Siciliano:** Writing – review & editing, Writing – original draft, Visualization, Project administration, Methodology, Investigation, Formal analysis, Data curation, Conceptualization. **Nicholas D. Henkel:** Writing – review & editing, Investigation, Conceptualization. **William G. Ryan V:** Writing – review & editing, Visualization, Software, Formal analysis, Data curation. **Ali Sajid Imami:** Formal analysis, Data curation. **John M. Vergis:** Writing – review & editing. **Chongchong Xu:** Methodology. **Taylen O. Arvay:** Visualization. **Smita Sahay:** Writing – review & editing, Methodology. **Priyanka Pulvender:** Methodology. **Abdul-rizaq Hamoud:** Investigation. **Chadwick Hales:** Resources. **Robert E. McCullumsmith:** Writing – review & editing, Supervision, Project administration, Funding acquisition, Conceptualization. **Zhexing Wen:** Writing – review & editing, Supervision, Project administration, Funding acquisition, Conceptualization.

## Declaration of competing interest

The authors declare that they have no known competing financial interests or personal relationships that could have appeared to influence the work reported in this paper.

## Data Availability

Data will be made available on request.
