# Supplementary material for: Proinflammatory transcriptomic and kinomic alterations in astrocytes derived from patients with familial Alzheimer's disease
Source: Brain Behav Immun Health. 2025 Jun 21;47:101044. doi: 10.1016/j.bbih.2025.101044 (PMC12246863; doi:10.1016/j.bbih.2025.101044)
Supplement: Multimedia component 1 [file mmc1.docx]

**Pro-Inflammatory Transcriptomic and Kinomic Alterations in Astrocytes Derived from Patients with Familial Alzheimer's Disease | Supplementary Materials**

**Supplementary Methods**

**1. 3PodR**

**1.1. Full Gene Set Pathway Analysis**

Gene Set Enrichment Analysis (GSEA) was conducted utilizing the complete set of genes and the fgsea R package [1]. Enrichment map gene sets were sourced from baderlab.org/EM_Genesets/, as this contains the most up-to-date annotations, and were employed for the analysis. The ranking metric was defined by [(log_2_-fold change (LFC) * (-log_10_(p-value)]. The leading-edge genes, extracted from significantly up- and down-regulated pathways (FDR < 0.05), were further ranked based on their frequency.

**1.2. Top Gene Pathway Analysis**

Over-Representation Analysis (ORA) was performed utilizing a set of the most significantly altered upregulated or downregulated genes (FDR < 0.05) and Enrichr [2]. The top gene set was be selected based on user-defined cut-offs of LFC, p-value, or false discovery rate (FDR) scores. The standard gene set databases are Gene Ontology, KEGG, Reactome, and MSigDB, just to name a few; however, this pipeline offers end-users the flexibility to explore gene set databases of their choice.

**1.3. Identification of Chemical Perturbagens using iLINCS**

Chemical perturbagens, in the context of iLINCS and LINCS, are small molecules or compounds that are used to induce a perturbation (e.g., changes in gene expression, protein levels, or other cellular responses) in cellular systems for experimental purposes. In the present study, the log_2_-transformed LFC and p-value for the 978 landmark genes in the LINCS L1000 project were extracted and submitted to iLINCS (<http://www.ilincs.org/ilincs/signatures/main/upload>) to acquire a list of the chemical perturbagens that alter the expression of genes of interest. The chemical perturbagens were clustered by mechanism of action (MOA) categories. The L1000 fireworks display application and the DrugBank database were utilized to obtain the MOA for each chemical perturbagen and display an interactive visualization of the small-molecule induced gene expression signatures.

**1.4. Report Generation**

Analysis and visualization of transcriptomic data was accomplished with 3PodReports, a method which performs and integrates GSEA, enrichR, and iLINCS results [3].

**2. PamGene Kinome Array**

**2.1. Background**

The PamGene platform is an established and extensively cited microarray technology that is renowned for its effectiveness in multiplex kinase activity profiling [4-7]. Kinases are vital regulatory proteins involved in numerous biological processes (e.g., cell cycle control, signal transduction, gene expression). Profiling kinase activity gives investigators insight into the post-translational modifications (e.g., phosphorylation) that regulate kinase activity and thus, best represents the functional state of the kinase in the cell [8]. Often, dysregulations in phenotypes may be due to more than one kinase, as several kinases may activate the same downstream target [9]. Therefore, to gain a comprehensive understanding of the role of kinase networks in a particular biological process, multiple kinases must be screened simultaneously. The PamStation12 microarray platform, utilizing the PamChip4 serine-threonine (STK) and phospho-tyrosine (PTK) microarray chips, have been developed for this purpose.

**2.2. Hardware: PamStation12 and PamChip4**

The PamGene PamStation12 Kinome Array is a high-throughput peptide array-based platform that enables multiplexed kinase activity profiling and facilitates the unbiased detection of kinase activity via the STK and/or the PTK microarray chips [4-7, 10, 11]. The basic design of the two chips is identical except the individual reporter peptides printed on the chips.

The PamChip4 STK and PTK chips contain 144 and 196 reporter peptides that are known substrates for serine/threonine and tyrosine kinases, respectively. Each chip contains four wells and each well contains approximately 300,000 copies of each peptide. The PamStation12 instrument can run three chips at once and thus, up to 12 samples may be run simultaneously on the array platform.

The PamStation12 instrument detects phosphorylation in real time. This information is used to directly measure altered kinase activity. For example, phosphorylation by the enzyme protein kinase A on the PamChip4 STK chip is concordant with its activity in solution [6]. After several cycles of sample injections and washes, a fluorescent antibody is applied to the PamChip4 PTK chip against the phosphorylated residues. The PamChip4 SKT chip requires two different antibodies to achieve fluorescence. Finally, the fluorescence levels may be visualized, where the intensity of the fluorescence is directly correlated with the extent of reporter peptide phosphorylation (i.e., activity levels).

The full PamGene platform workflow is detailed in Supplementary Figure 1 (S1).

**2.3. Chip Coverage**

The PamGene instrument provides coverage for a substantial portion of the human kinome, enabling a comprehensive analysis of kinase activity. Of the approximately 500 kinases that comprise the human genome [12, 13], 245/376 (65%) serine/threonine and 89/93 (96%) tyrosine kinases may be mapped to the STK and PTK chips, respectively. Furthermore, the platform can detect approximately 18/21 (86%) dual specificity kinases, covering about 72% of the entire kinome. Taken together, these findings demonstrate the capability of the PamGene platform to detect altered kinase activity efficiently and accurately, which ultimately provides insight into numerous molecular processes within the biological system being investigated.

**2.4. Data Generation**

Data generation for the PamGene platform involves three distinct steps: the Bench step, the Imaging Step, and the Preprocessing step.

*The Bench Step* is when investigators acquire and prepare the samples from subjects of interest for analysis on the PamChip4. The standard sample preparation protocols are provided by the PamGene Corporation (<https://pamgene.com/ps12/>). These standardized procedures ensure reproducibility and accuracy of the results. Briefly, samples are treated with protease and phosphatase inhibitors to control the catalytic activity and stability of kinases.

*The Imaging Step* is when images of the fluorescent activity on the chip are generated. During this step, the sample and reagent mixtures are added to the Pamchip4 chip and the Evolve 3 kinetic image capture software is utilized to take photographs.

Standardized imaging protocols are provided by the PamGene corporation. For the PamChip4 PTK chip, the process is as follows: Chips are loaded with the processed sample mixture from the Bench step. Blocking buffer and the PamGene reagent mix that includes the fluorescent antibody (allowing for peptide phosphorylation and activity detection) are sequentially added. After a two-minute processing time, peptide phosphorylation is monitored via images that are captured every 5 minutes for a duration of 60 minutes at exposure lengths of 5 milliseconds, 25 milliseconds, and 100 milliseconds, allowing real time recording of the reaction kinetics. Once all cycles are complete, a final image is captured at the following exposure lengths: 5 milliseconds, 25 milliseconds, 50 milliseconds, and 100 milliseconds.

The standardized protocols provided by the PamGene Corporation include internal control tests and normalization strategies to help minimize technical variation and ensure the sensitivity and reliability of the generated data. The technical variation between chips and runs is reported as <9% and <15%, respectively, as determined by the coefficient of variability. To account for technical variation between runs as well as to normalize the data, an internal control sample may be added.

*The Preprocessing Step* is the final stage of data generation, where images are preprocessed to quantify the activity of each peptide via fluorescence levels. Briefly, the PamGene BioNavigator software is utilized to capture and quantify the fluorescence values (<https://pamgene.com/wp-content/uploads/2020/09/BioNavigator-User-Manual-vs2.3-2020.pdf>). Annotation data is added to the extracted fluorescence value to allow for the identification of data at the various exposure levels for each sample, thereby accurately displaying the resolution of each image. A “Signal – Background” metric of intensity is calculated that accounts for the background signal intensity. A “Signal Saturation” value is also measured for each well on the chip. The final data is displayed in an output table that may be further processed by downstream software packages.

The dynamic range of raw signal intensities is typically between the values of 0 – 3,000. The linear regression slope of the signal intensity, averaged across the biological replicates, is interpreted as a function of exposure time and is used to both increase the dynamic range of the measurements and represent the peptide phosphorylation intensity for downstream comparative analyses. The signal intensity ratio between the case and control samples is used to calculate fold change (FC) values. Peptides with a FC of at least 15% (i.e., FC >1.15 or FC <0.85) are considered differentially phosphorylated. This threshold has been chosen based on previous reports that suggest small changes in kinase activity are sufficient to trigger biologically relevant changes [10, 11, 14].

Prior to proceeding to activity analysis, all inactive peptides, identified as having a raw signal of ≤5 or an R^2^ of <0.90 during the linear regression, are removed. Values at these thresholds deem the kinase undetectable or non-linear in the post-wash phase.

**2.5. Assessment of Upstream Kinases**

The raw output from the preprocessing step indicates the phosphorylation status of the individual peptides on the chip. As these results are a measure of the peptides, or targets, that kinases act on, the collected data are considered an indirect measure of kinase activity. Peptides on the array, and in general, may be phosphorylated by more than one kinase. Therefore, individual kinases upstream of a specific peptide may also be involved in the phosphorylation of such targets, and identification of these kinases is imperative.

Upstream kinase identification begins by selecting a particular state of phosphorylation from the input data, usually the data from the final cycle of the array run. For each sample, a variable is defined that will summarize the phosphorylation level of that peptide. The change in phosphorylation status for this group is compared to a control group. Finally, a combination of experimental, literature-based, and computational predictions is utilized to identify the most prominent upstream kinases.

Lastly, four different software packages exist that may be deployed for upstream kinase identification: upstream kinase analysis, kinome random sampling analysis, kinase enrichment analysis v3, and post-translational modification signature enrichment analysis. All these packages rely, to varying extents, on publicly available mapping databases and are discussed in detail below.

**2.6. Upstream Kinase Analysis (UKA)**

This package was developed by the PamGene Corporation (s’-Hertengobosch, Netherlands). The recommended, standardized protocol for UKA is integrated into the manufacturer’s BioNavigator software methods. Briefly the UKA method relies on a curated database of kinase substrate interactions created by the PamGene Corporation. UKA processes the raw output from the PamStation12 instrument by filtering low intensity peptides and scaling/normalizing the entire dataset, so the values fall in the range of 0 – 100. The program then calculates a “Kinase Score” for each kinase and reports the kinases that receive the highest scores.

Some advantages of the UKA package are that it provides the results for specific kinases (as opposed to families) and that it possesses a low false positive rate compared to other packages. One putative limitation of the UKA package is that its thresholds for specific variables may be too stringent for discovery-based experiments.

**2.7. Kinome Random Sampling Analyzer (KRSA)**

This package was developed by the Cognitive Disorders Research Laboratory (CDRL) at The University of Toledo College of Medicine and Life Sciences (UTCOMLS), led by Dr. Robert McCullumsmith, M.D., Ph.D. [15].

This algorithm utilizes data derived from the kinome array and the mapping from the PamChip4 file. Peptides are selectively filtered based on specified advancement criteria, including the signal intensity at maximum exposure time and the R^2^ value from the linear regression model. The algorithm then generates a list of filtered peptides, which is subsequently employed in the subsequent analysis step.

*Curation of the Database of Upstream Kinases*. KRSA relies on a curated database of upstream kinases for the peptides present on the array. Protein kinases predicted to act on phosphorylation sites within the peptide sequences on the PamChip4 chip are identified using the GPS 3.0 and Kinexus Phosphonet (Kinexus Bioinformatics) programs [16-18]. These programs provide predictions for the kinases that may be targeting peptide sequences, where the output kinases are ordered by the likelihood of binding. The union of the highest ranked five kinases in Kinexus and kinases with scores more than 2X the prediction threshold in GPS 3.0 are considered predicted kinases for each peptide and are utilized in the KRSA analysis [6]. This list is combined with kinases shown in the literature to act on the phosphorylation sites of the peptides via PhosphoELM (<http://phospho.elm.eu.org>) and PhosphoSite Plus (<https://www.phosphosite.org>).

*Empirical Measures of Statistical Significance*. KRSA performs a permutation analysis (utilizing the Monte Carlo simulation) by taking a random sample of the same number of observed peptides that passed the advancement criteria. For each simulation, upstream kinase counts are accumulated providing a standard normal distribution for the number of times a kinase would be predicted to be assigned by chance alone. The simulation is run 2,000 times, yielding a distribution histogram as well as median, mean, and standard deviation values for specific kinases. A comparison is made between the observed (i.e., the number of times a kinase mapped to the peptide from the actual experiment that passed quality control and FC thresholds) and the expected (i.e., from the permutation analysis) kinases. Observed kinases are assigned a z-score value, generated based on the number of standard deviations the value is from the mean of the expected upstream kinases. A positive z-score suggests over-involvement (NOT higher activity) of this kinase in the substrate studied versus in the control group. Since the z-score signifies statistical significance (i.e., outside of a pre-determined confidence interval), we consider any kinase with a z-score of >2 to be a “hit,” warranting further experimental assessment. In some cases, we adjust the z-score threshold by lowering the stringency to increase the number of candidate kinases for discovery-based studies.

*Presentation of the data in KRSA: Heatmaps*. The KRSA heatmaps are generated from the signal intensity data. The selection of peptides is based on the quality control criteria explained above. The values on the heatmap are the linear regression slope of the signal intensity, which may be interpreted as a function of exposure time and represents the peptide phosphorylation intensity.

*Presentation of the Data in KRSA: Violin Plots*. The KRSA violin plots showcase the distribution of the signal intensity of significant peptides on a per-group basis.

*Presentation of the Data in KRSA: Waterfall Plots*. The KRSA waterfall plots are generated from the z-score values for each kinase. Briefly, the z-score for each kinase is generated on a chip-by-chip basis and averaged across the three chips utilized in one assay run. The waterfall plot displays the distribution of z-scores for each chip. The red dot displayed is the mean z-score value.

The KRSA package was developed to complement the PamGene Corporation’s UKA package. KRSA possesses a less stringent strategy to identify and assign upstream kinases, providing more putative “hits” for discovery-based studies, where findings will be confirmed with additional studies. One limitation of KRSA is that it only provides kinase families as output, as opposed to specific kinases. We often combine KRSA and UKA analyses, in this order, to determine a hit “family” then identify the specific family members with UKA or another package (such as KEA3, below).

**2.8. Kinase Enrichment Analysis 3 (KEA3)**

KEA3 is an upstream kinase identification and assignment method developed by the Ma’ayan laboratory (<https://maayanlab.cloud/kea3/>) that relies on the known kinase protein interactions, kinase substrate interaction data, and associated co-expression and co-occurrence data [19]. The KEA3 web application takes a list of differentially expressed phosphorylated proteins and their associated FC values as input. Utilizing gene set libraries from kinase-substrate interaction databases, KEA3 outputs a ranked list of the top predicted kinases displayed as networks, subnetworks, bar graphs, and a cluster-gram.

**2.9. Integration of Upstream Kinase Assignments Across Packages**

The tools above utilize various, independent methods to identify upstream kinases. This necessitates the use of an integration system to identify consensus upstream kinases across datasets. For this purpose, we utilize the software Creedenzymatic [22]. The Creedenzymatic program processes the results from at least two of the four possible upstream kinase identification analysis methods and generates a consensus figure with kinases deconvolved and ranked based on their presence in the results. The integration of these packages ultimately allows the investigator to identify and select dysregulated kinases for further analysis and confirmation studies.

**3. Differential Gene Expression (DGE) Analysis**

**3.1. Generating the Differential Genes**

Transcript abundances were quantified from paired-end reads (raw FASTQ files) against the *Homo sapiens* reference transcriptome (hg19) using kallisto (version 0.43.1) [23]. Transcriptome-wide gene counts were obtained through gene level transcript summarization with the tximport R package (version 1.26.1) [24]. DGE analysis was performed after filtering for low-expression genes with the *filterByExpr* function using the edgeR R package (version 3.40.2) [25].

**3.2. Heatmap and Volcano Plot Generation**

Expression profiles of the top 500 most variable genes (defined by the greatest sample variance of the scaled and centered log-transformed Trimmed Mean of M-values (TMM)-normalized counts per million) were visualized using the ComplexHeatmap R package (version 2.14.0) [26]. Differential expression profiles were visualized with standard volcano plots. The difference in gene expression (LFC) between experimental comparisons was plotted against the significance values (-log_10_p-value). The top ten genes that showed the greatest change (|LFC|) were labelled. The dashed lines indicate the significance threshold of adjusted p-values < 0.05 and absolute log2 fold change values > 1.

**4. Network-based Multiomics Integration of Transcriptome and Kinome**

**4.1. Uncovering Dysregulated Pathways and Hub Nodes**

The Kinograte R package [27], which implements an optimized version of the well-established PCSF algorithm [28], was utilized to generate an integrated multiomic protein-protein interaction (PPI) network consisting of transcriptomic “hits” (FDR<0.05) and kinomic “hits” returned from Creedenzymatic analysis. Node prizes were assigned by percentile rank of respective LFC or mean kinome rank and edge costs by inverse STRING-DB [29] interaction confidence. PPI network nodes were then ranked by the average of their node prize and eigen-centrality as input for gene-set over-representation analysis using the fgsea R package [1] with the Gene Ontology database [30]. Dysregulated pathways (FDR < 0.05) were used to generate subnetworks to visualize the interactions between genes from significant pathway results.

**4.2. Functional Interpretation of Dysregulated Pathways**

Dysregulated pathways (FDR < 0.05) were subjected to functional clustering and visualization using PAVER [31], a meta-clustering algorithm tailored for pathways of interest. PAVER employs a meta-clustering strategy to identify the most representative terms (MRTs) for hierarchically clustered pathway embeddings [32]. This is achieved by selecting the term that exhibits the highest cosine similarity to its respective cluster’s average embedding. Subsequently, UMAP scatter plots representing individual pathways were created, with colors indicating their assigned cluster and shapes denoting the experimental comparison they originated from.


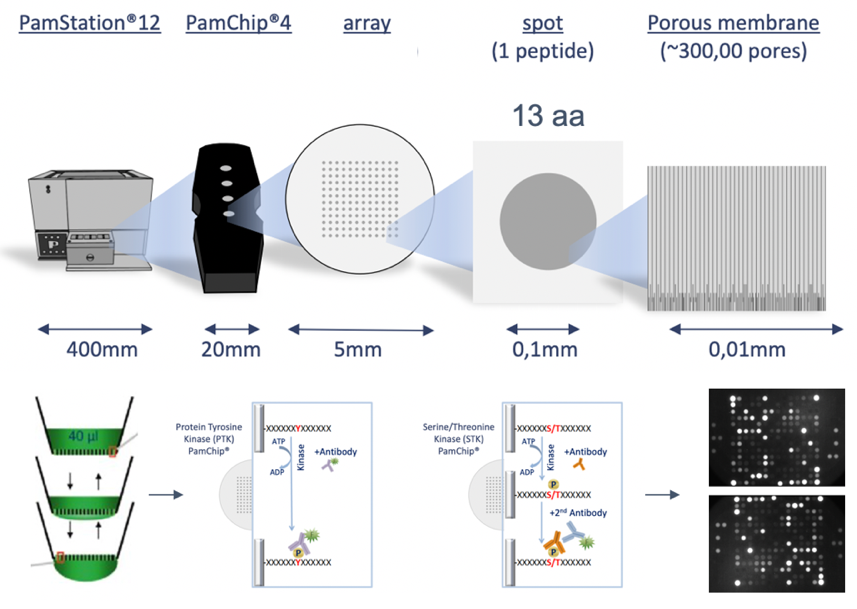
**Supplementary Figure 1 (S1):**

**Figure S1: The PamGene Platform Workflow**. The PamGene PamStation12 Kinome Array is a high-throughput peptide array-based platform that enables multiplexed kinase activity profiling and facilitates the unbiased detection of kinase activity via the serine/threonine kinase (STK) and/or the phosphor-tyrosine kinase (PTK) microarray chips. The PamChip4 PTK chip utilized in this study contains 196 reporter peptides known to be substrates for tyrosine kinases. The PamChip4 STK chip contains 144 reporter peptides known to be substrates for serine/threonine kinases. Each chip contains four wells and each well contains approximately 300,000 copies of each peptide. The PamStation12 instrument can run three chips at once and thus, up to 12 samples may be run simultaneously on the array platform. The PamStation12 instrument detects phosphorylation in real time. After several cycles of sample injections and washes, a fluorescent antibody is applied to the PamChip4 chip against the phosphorylated residues. The fluorescence levels may be visualized, where the intensity of the fluorescence is directly correlated with the extent of reporter peptide phosphorylation (i.e., activity levels). *This figure was reprinted with permission from PamGene International B.V.*

**References**

1. Korotkevich, G., et al., *Fast gene set enrichment analysis.* bioRxiv, 2021: p. 060012.

2. Chen, E.Y., et al., *Enrichr: interactive and collaborative HTML5 gene list enrichment analysis tool.* BMC Bioinformatics, 2013. **14**: p. 128.

3. Ryan, W., *willgryan/3PodR_bookdown: Pre-release to generate DOI. Zenodo. Available:* [*https://zenodo.org/records/8190833*](https://zenodo.org/records/8190833)*.*

4. Arsenault, R., P. Griebel, and S. Napper, *Peptide arrays for kinome analysis: new opportunities and remaining challenges.* Proteomics, 2011. **11**(24): p. 4595-609.

5. Baharani, A., et al., *Technological advances for interrogating the human kinome.* Biochemical Society Transactions, 2017. **45**(1): p. 65-77.

6. Bentea, E., et al., *Kinase network dysregulation in a human induced pluripotent stem cell model of DISC1 schizophrenia.* Mol Omics, 2019. **15**(3): p. 173-188.

7. Hilhorst, R., et al., *Peptide microarrays for detailed, high-throughput substrate identification, kinetic characterization, and inhibition studies on protein kinase A.* Analytical Biochemistry, 2009. **387**(2): p. 150-161.

8. Mann, M. and O.N. Jensen, *Proteomic analysis of post-translational modifications.* Nat Biotechnol, 2003. **21**(3): p. 255-61.

9. Zhang, J., P.L. Yang, and N.S. Gray, *Targeting cancer with small molecule kinase inhibitors.* Nat Rev Cancer, 2009. **9**(1): p. 28-39.

10. McGuire, J.L., et al., *Abnormalities of signal transduction networks in chronic schizophrenia.* NPJ Schizophr, 2017. **3**(1): p. 30.

11. Dorsett, C.R., et al., *Traumatic Brain Injury Induces Alterations in Cortical Glutamate Uptake without a Reduction in Glutamate Transporter-1 Protein Expression.* J Neurotrauma, 2017. **34**(1): p. 220-234.

12. Manning, G., et al., *The protein kinase complement of the human genome.* Science, 2002. **298**(5600): p. 1912-1934.

13. Manning, G., *Genomic overview of protein kinases.* WormBook : the online review of C. elegans biology, 2005: p. 1-19.

14. Appuhamy, J.A., et al., *Effects of AMP-activated protein kinase (AMPK) signaling and essential amino acids on mammalian target of rapamycin (mTOR) signaling and protein synthesis rates in mammary cells.* J Dairy Sci, 2014. **97**(1): p. 419-29.

15. DePasquale, E.A.K., et al., *KRSA: An R package and R Shiny web application for an end-to-end upstream kinase analysis of kinome array data.* PLOS ONE, 2021. **16**(12): p. e0260440.

16. Xue, Y., et al., *GPS 2.1: enhanced prediction of kinase-specific phosphorylation sites with an algorithm of motif length selection.* Protein Engineering Design and Selection, 2011. **24**(3): p. 255-260.

17. Wang, C., et al., *GPS 5.0: An Update on the Prediction of Kinase-specific Phosphorylation Sites in Proteins.* Genomics, Proteomics & Bioinformatics, 2020. **18**(1): p. 72-80.

18. Xue, Y., et al., *GPS: a comprehensive www server for phosphorylation sites prediction.* Nucleic Acids Research, 2005. **33**(suppl_2): p. W184-W187.

19. Kuleshov, M.V., et al., *KEA3: improved kinase enrichment analysis via data integration.* Nucleic Acids Res, 2021. **49**(W1): p. W304-W316.

20. Krug, K., et al., *A Curated Resource for Phosphosite-specific Signature Analysis.* Mol Cell Proteomics, 2019. **18**(3): p. 576-593.

21. Cox, J. and M. Mann, *MaxQuant enables high peptide identification rates, individualized p.p.b.-range mass accuracies and proteome-wide protein quantification.* Nat Biotechnol, 2008. **26**(12): p. 1367-72.

22. Khaled, A.S. Imami, and J. Creeden, *CogDisResLab/creedenzymatic: v 5.0.0 Version Reset*. 2022, Zenodo.

23. Bray, N.L., et al., *Near-optimal probabilistic RNA-seq quantification.* Nature Biotechnology, 2016. **34**(5): p. 525-527.

24. Soneson, C., M. Love, and M. Robinson, *Differential analyses for RNA-seq: transcript-level estimates improve gene-level inferences [version 1; peer review: 2 approved].* F1000Research, 2015. **4**(1521).

25. Robinson, M.D., D.J. McCarthy, and G.K. Smyth, *edgeR: a Bioconductor package for differential expression analysis of digital gene expression data.* Bioinformatics, 2010. **26**(1): p. 139-40.

26. Gu, Z., R. Eils, and M. Schlesner, *Complex heatmaps reveal patterns and correlations in multidimensional genomic data.* Bioinformatics, 2016. **32**(18): p. 2847-2849.

27. Alganem, K., *Network-Based Integration of Multi-Omics Datasets*. 2022, University of Toledo Health Science Campus.

28. Akhmedov, M., et al., *PCSF: An R-package for network-based interpretation of high-throughput data.* PLOS Computational Biology, 2017. **13**(7): p. e1005694.

29. von Mering, C., et al., *STRING: known and predicted protein-protein associations, integrated and transferred across organisms.* Nucleic Acids Res, 2005. **33**(Database issue): p. D433-7.

30. Ashburner, M., et al., *Gene ontology: tool for the unification of biology. The Gene Ontology Consortium.* Nat Genet, 2000. **25**(1): p. 25-9.

31. Ryan, W., *willgryan/PAVER: Pre-release to generate DOI*. Zenodo.

32. Edera, A.A., D.H. Milone, and G. Stegmayer, *Anc2vec: embedding gene ontology terms by preserving ancestors relationships.* Briefings in Bioinformatics, 2022. **23**(2).
